# Multiparty weighted threshold quantum secret sharing based on the Chinese remainder theorem to share quantum information

**DOI:** 10.1038/s41598-021-85703-7

**Published:** 2021-03-17

**Authors:** Yao-Hsin Chou, Guo-Jyun Zeng, Xing-Yu Chen, Shu-Yu Kuo

**Affiliations:** 1 Department of Computer Science and Information Engineering, National Chi Nan University, Puli, 54561 Taiwan; 2 Department of Computer Science and Engineering, National Chung Hsing University, Taichung, 402 Taiwan

**Keywords:** Applied mathematics, Quantum information, Qubits

## Abstract

Secret sharing is a widely-used security protocol and cryptographic primitive in which all people cooperate to restore encrypted information. The characteristics of a quantum field guarantee the security of information; therefore, many researchers are interested in quantum cryptography and quantum secret sharing (QSS) is an important research topic. However, most traditional QSS methods are complex and difficult to implement. In addition, most traditional QSS schemes share classical information, not quantum information which makes them inefficient to transfer and share information. In a weighted threshold QSS method, each participant has each own weight, but assigning weights usually costs multiple quantum states. Quantum state consumption will therefore increase with the weight. It is inefficient and difficult, and therefore not able to successfully build a suitable agreement. The proposed method is the first attempt to build multiparty weighted threshold QSS method using single quantum particles combine with the Chinese remainder theorem (CRT) and phase shift operation. The proposed scheme allows each participant has its own weight and the dealer can encode a quantum state with the phase shift operation. The dividing and recovery characteristics of CRT offer a simple approach to distribute partial keys. The reversibility of phase shift operation can encode and decode the secret. The proposed weighted threshold QSS scheme presents the security analysis of external attacks and internal attacks. Furthermore, the efficiency analysis shows that our method is more efficient, flexible, and simpler to implement than traditional methods.

## Introduction

Secret sharing is a basic and essential cryptography protocol. The dealer can divide the secret into many shares and sends shares to different agents. Only when these authorized agents collaborate can reconstruct the original secret. Conversely, unauthorized users cannot complete this task. However, if one or more agents cannot get together for some reason, or the dealer wants to give different agents different weights of shares, the secret sharing protocol should be more flexible to solve problems in different scenarios such as data repair, hierarchical structures, and financial infidelity, etc. The threshold secret sharing allows shares to reconstruct the secret when the number of shares is greater than or equal to the threshold value *t*. In application, it allows some involved computers to reconstruct the important data if others involved in the scheme are destroyed. The weighted threshold secret sharing allocates *n* agents or machines a respective weight $(w _{1},w _{2},\ldots ,w _{n})\in w$. When the sum of weights of agents who cooperate together is greater than or equal to the weighted threshold value $\omega$, they can successfully reconstruct the secret message. In application, it ensures a stable system. Let every user or machine have its own weight according to different levels. It is important that the high weighted individuals have higher authority than the low weighted individuals in a hierarchical structure. Therefore, users with high authority can complete something easily. Conversely, users with low authority can only decide something with the help of a user with higher authority or more users with low authority.

The security of traditional cryptography is based on computational complexity. With the advent of quantum algorithms in 1997^1^, quantum computers began using algorithms to achieve parallel computations that were based on physics law, make them incredibly quickly crack RSA (Ron Rivest, Adi Shamir, and Leonard Adleman), AES (Advanced encryption standard), and protocols based on RSA and AES security, which are all based on mathematical complexity. With the development of quantum cryptography^2^ which based on physical law can achieve unconditionally secure^3–7^. As a result, quantum cryptography has attracted research attention and become widely used in data transmission and information security. Quantum secret sharing (QSS) has been developed firstly by Hillery et al.^8^ in 1999, they built QSS with Greenberger-Horne-Zeilinger (GHZ) states, which inspired numerous studies afterward^9,10^. However, most studies use traditional methods such as Lagrange Interpolation to build quantum secret sharing schemes, which focuses on the distribution of classical bits as shares^11,12^ instead of sharing quantum bits. Hence, this study focus on sharing the quantum information with Chinese Remainder Theorem (CRT), because CRT can use different coprime divisors as the respective weight of the agents (unlike Lagrange Interpolation).

Quantum secret sharing is more difficult than with classical information. Thus, most proposed schemes for sharing secrets use classical information^10–12^ not quantum information^13–16^. Moreover, $(w , \omega , {n})$-weighted threshold quantum secret sharing scheme are more difficult both than (*n*, *n*)-quantum secret sharing and (*t*, *n*)-threshold quantum secret sharing schemes. The complication of $(w , \omega , {n})$-weighted threshold QSS makes them extremely difficult to successfully implement because most proposed schemes cannot use quantum states to distribute their weights fairly. Regarding (*t*, *n*)-threshold secret sharing schemes, the first threshold quantum secret sharing scheme based on a multi-dimensional Hilbert space^13^ was proposed in 1999. Tokunaga et al.^14^ proposed a threshold method using the Lagrange Interpolation formula. However, Lagrange Interpolation is not efficient and flexible enough to construct a weighted scheme, and the number of transmissions it spends increases with the weight. Therefore, Iftene et al.^15^ proposed using the CRT to share quantum information. In 2015, Qin et al.^16^ constructed a (*t*, *n*)-threshold quantum secret sharing schemes using the phase shift operation.

Many quantum threshold secret sharing protocols have been proposed. Most researchers try to build that based on error correction or the way traditional methods to turn quantum scheme, but it is still very difficult. Therefore, they hope to achieve quantum properties and share quantum states. The traditional method of Lagrange Interpolation is an extensive approach. It is difficult to achieve only by Lagrange interpolation. It is clear that both schemes are difficult to construct, and weighted threshold schemes are more difficult to build than threshold schemes. To the best of our knowledge, there is no significant study in the quantum field has built a $(w , \omega , {n})$-weighted threshold quantum secret sharing scheme yet, this study presented a novel method based on the CRT and phase shift operation to share quantum information and build a $(w , \omega , {n})$-weighted threshold quantum secret sharing scheme. CRT’s characteristics of dividing and recovery make it simply distributes shares and reconstructed secret. The reversibility of phase shift operation can revert the quantum states of the encoded secrets. Therefore, the proposed method is able to build a weighted threshold QSS scheme and share the quantum information using the CRT and phase shift operation. The dealer divides the secret/key into *n* partial keys and distributes to every participant a share as a private key by quantum secure direct communication (QSDC)^18^ according to the weight of each participant. Next, the dealer uses the phase shift operation to encode a quantum state with the key and then sends the quantum state to each participant. When the sum of the participants’ weights achieves $\omega$, every participant will be able to perform the inverse phase shift operation one by one with CRT. The participants can then cooperate to reconstruct the secret and obtain quantum information. Conversely, if it cannot meet the above condition, the participants will be unable to cooperate to obtain the quantum information. In addition, in order to detect eavesdroppers attempting to steal quantum particles when the dealer and participants transfer particles in a quantum channel, some decoy particles are inserted into the quantum sequence. Eavesdroppers can be detected by the measurement result, thereby building an unconditional security quantum channel. The proposed method not only can implement simpler than other traditional methods but also achieves unconditional security.

With the rapid development of the quantum computers^19–27^, IBM now provides remote access to their quantum computers. People can use IBM Q experiences to learn quantum computation such as building quantum circuits and simulating some quantum algorithms. We have a registered IBM Q system account and have tested some tasks such as the Deutsch-Jozsa and Shor’s algorithm. However, IBM Q service mostly focuses on simulating quantum algorithms and circuits in a very small scale and does not provide multiple quantum computers and channels to simulate quantum networks. Nevertheless, in recent years, there are many outstanding researchers investigating the concept of quantum internet^28–37^ showing that the future of quantum networks is very promising. We have checked and simulated the proposed QSS protocol, and there is no doubt it will become a great secret sharing protocol and can be perfectly suited for large-scale quantum internet applications in the future.

## Results

This section consists of three subsections, including the preliminaries, the proposed protocol, and its security and efficiency analyses. The preliminaries introduce phase shift operations, Lagrange interpolation, and Weighted Threshold Secret Sharing Based on CRT. Then, the proposed protocol is introduced step by step. Finally, security and efficiency analyses are presented.

### Preliminaries

This subsection introduces the main related knowledge and preliminaries, including phase shift operations explain how to change the quantum state. Lagrange Interpolation and weighted threshold secret sharing based on Chinese remainder theorem (CRT) explain what CRT is and how to use it to build the threshold and weighted threshold schemes.

#### Phase shift operations

According to quantum theory, quantum states can be changed by unitary operations. The phase shift operation is a kind of unitary operation as expressed in Eq. (1), that has additive and commutative properties. It can perform $U(\theta )$ to change the quantum state and then perform inverse $U(-\theta )$ to revert the quantum state.

1 $$\begin{aligned} \begin{aligned} U(\theta )=\begin{bmatrix} cos(\theta ) &{} -sin(\theta )\\ sin(\theta )&{} ~~cos(\theta ) \end{bmatrix} \end{aligned} \end{aligned}$$

#### Lagrange interpolation

Lagrange Interpolation uses multiple points to build line segments in the same condition. Lagrange Interpolation has many applications in communication and computer science. In cryptography, researchers have proposed many encoding and decoding methods using Lagrange Interpolation. Several versions of Lagrange Interpolation have also been proposed^13,14,16^. The principle of the Lagrange Interpolation method is that any different $n + 1$ or more points can be used to reconstruct the only polynomial function of *n* degree. For example, in the Lagrange Interpolation method, it needs at least three points to construct a polynomial function of 2 degrees. The advantages of the Lagrange Interpolation method are that it is easy to use points to construct functions and easy to build threshold schemes. However, it is not efficient and not flexible to build weighted schemes and the number of transmissions increases with the weight.

#### Weighted threshold secret sharing based on CRT

The principle of the CRT method is that any *n* coprime divisor and corresponding remainder can be used to reconstruct the number with the same conditions. The advantages of CRT are that it is easy to use the divisor and remainder to build a number and it is efficient and flexible to build weighted schemes. Therefore, the CRT is more flexible than Lagrange Interpolation in weighted threshold secret sharing because an *n* coprime divisor is taken as the respective weight of the users. The larger the weight, the larger the divisor. However, Lagrange Interpolation differs in that it cannot use point numbers of magnitude for the weight of the user. Lagrange Interpolation uses multiple different points to express the weights of users, which is inefficient. The proposed weighted threshold secret sharing scheme is based on the CRT scheme^15^. But, the range of remainder *S* differs from that of the threshold scheme. When the weight of the circle can be given, the possible range of *S* will shrink, and will be closer to *S*. When the sum of the weight is greater than the threshold weighted value, the range of *S* can be determined and a more flexible weight threshold can be achieved. Therefore, it is necessary to determine the limited range of *S* according to the respective user weights. Then, *S* can be reconstructed if and only if the sum of the weights of the users is greater than or equal to a fixed weighted threshold.

### The proposed protocol

Most proposed quantum threshold schemes^13,14,16,17^ were based on a multi-dimensional quantum state and Lagrange Interpolation and are too complicated to implement practically. They are unable to fairly use the quantum states to distribute their weights and share quantum information. The proposed method is the first attempt to construct a $(w , \omega , n)$-weighted threshold QSS method sharing quantum information. The scheme is flexible that the dealer can decide the different weight of the shares and distributes these shares to each participant. The condition to reconstruct the secret is that calculating of all weights of participant who show up to cooperate, then when the sum of weight exceed the threshold $\omega$ set by dealer can find out the secret. The proposed method uses CRT, phase shift operations, and single quantum particles to build a $(w , \omega , n)$-weighted threshold QSS scheme. Based on the principle of CRT, the dealer divides the key into *n* partial keys and distributes these shares to participants. Each participant receives a corresponding private partial key, according to its own weight value (which is the greater the weight, the larger the share). The dealer then converts the key into radian $\theta$ and performs phase shift operation $U(\theta )$ on the secret to encrypt the quantum state. It is not necessary to have all participants cooperate, when the sum of the weights of the participants is equal to or more than the weighted threshold, the participants can reconstruct the secret. In other words, when participants who have greater weight, the secret can be reconstructed by a smaller number of participants. On the contrary, when participants who have lesser weights, the secret should be reconstructed by a large number of participants. Also, according to the principle of CRT participants can convert their own private partial key into radian $-\theta _{i}$ and perform inverse phase shift operation $-U(\theta )$ on the quantum state one by one to reconstruct and receive quantum information.

Based on the above description, a $(w , \omega , n)$-weighted threshold quantum secret sharing scheme with *n* participants works as follows. The dealer gives every participant $p_{i}$ a respective weight $w _{i}$ that is lower than weighted threshold $\omega$ for all $1\le i\le n$. Then, when the sum of the weights of the participants is equal to or greater than the weighted threshold $\omega$, the participants can cooperate to reconstruct the secret and receive the shared quantum information. Consider a scheme involving three participants (*A*, *B* and *C*), this section gives an example to describe our protocol. The dealer assigns their weights as $w _{1}=1$, $w _{2}=1$, and $w _{3}=2$, respectively, and sets the weighted threshold value $\omega =3$ in order to establish a ((1, 1, 2), 3, 3)-weighted threshold QSS scheme. According the principle of CRT, the participants are able to cooperate to reconstruct the secret using phase shift operation. The six steps to complete the protocol and an example are provided as follows.

**Step 1. The dealer sets private keys/ shares:** Based on CRT, the dealer decides the private keys and depending on the weight $w _{i}$ of each participant $p_{i}$, the dealer prepares the coprime positive integers $m_{i}$ and $gcd(m_{i},m_{j})=1$ for all $1<i<j<n$ to be respective private/partial keys for each participant. The function gcd ($m_{i}$, $m_{j}$) means finding the greatest common divisor (gcd) of two integers, $m_{i}$ and $m_{j}$. The positive integer $m_{i}$ consists of prime numbers and the value of $m_{i}$ is according to the weight value of each participant. When the weight value $w _{i}$ is low, it means that the positive integer $m_{i}$ will be constituted of a lower product of prime numbers. Conversely, if the weight value $w _{i}$ is high, it represents that the positive integer $m_{i}$ is made up of a higher product of prime numbers. Therefore, the weight $w _{1} \le w _{2} \le \cdots \le w _{n}$ and the corresponding different positive integer $m_{1}< m_{2}< \cdots < m_{n}$ are obtained. For example, the dealer sets *A*, *B*, and *C*’s respective weights as $w _{1}=1$, $w _{2}=1$, and $w _{3}=2$. This means that $m_{1}$ is made up of the product of 2, $m_{2}$ is made up of the product of 7, and $m_{3}$ is made up of the product of 3 and 5. Thus, the dealer sets $m_{1}=2$, $m_{2}=7$, and $m_{3}=15$.

**Step 2. The dealer decides the key:** Depending on the weight $w _{i}$ of each participant $p_{i}$ and the corresponding coprime positive integer $m_{i}$, the dealer calculates set *L*, and combines it with the product of $m_{i}$, where $\sum _{i\in n}w_{i}\le \omega -1$. This means that the sum of the weights is lower than threshold $\omega$. The maximum from set *L* is chosen to be positive integer *K*. Similarly, the dealer computes set *G*, and combines it with the product of $m_{i}$, where $\sum _{i\in n}w_{i}\ge \omega$. This means that the sum of the weights is greater than weighted threshold $\omega$. The minimum value from set *G* is selected as positive integer *Q*. Finally, the dealer can choose the random positive integer between *K* and *Q* and decide to be the key *S*. For example, after calculating the product of $m_{i}$, set *L* is $\{\{m_{1}\}, \{m_{2}\}, \{m_{1}, m_{2}\}\}$, which is equal to $\{\{2\}, \{7\}, \{14\}\}$, and set *G* is $\{\{m_{1}, m_{3}\},\{m_{2}, m_{3}\}, \{m_{1}, m_{2}, m_{3}\}\}$, which is equal to $\{\{30\}, \{105\}, \{210\}\}$. Thus, *K*, the maximum from set *L*, is $\{\{m_{1}, m_{2}\}\}$, which is equal to 14, while *Q*, the minimum value from set *G*, is $\{\{m_{1}, m_{3}\}\}$, which is equal to 30. Finally, the dealer can choose a key *S* between 14 and 30, and then decide to be 23.

**Figure 1:**
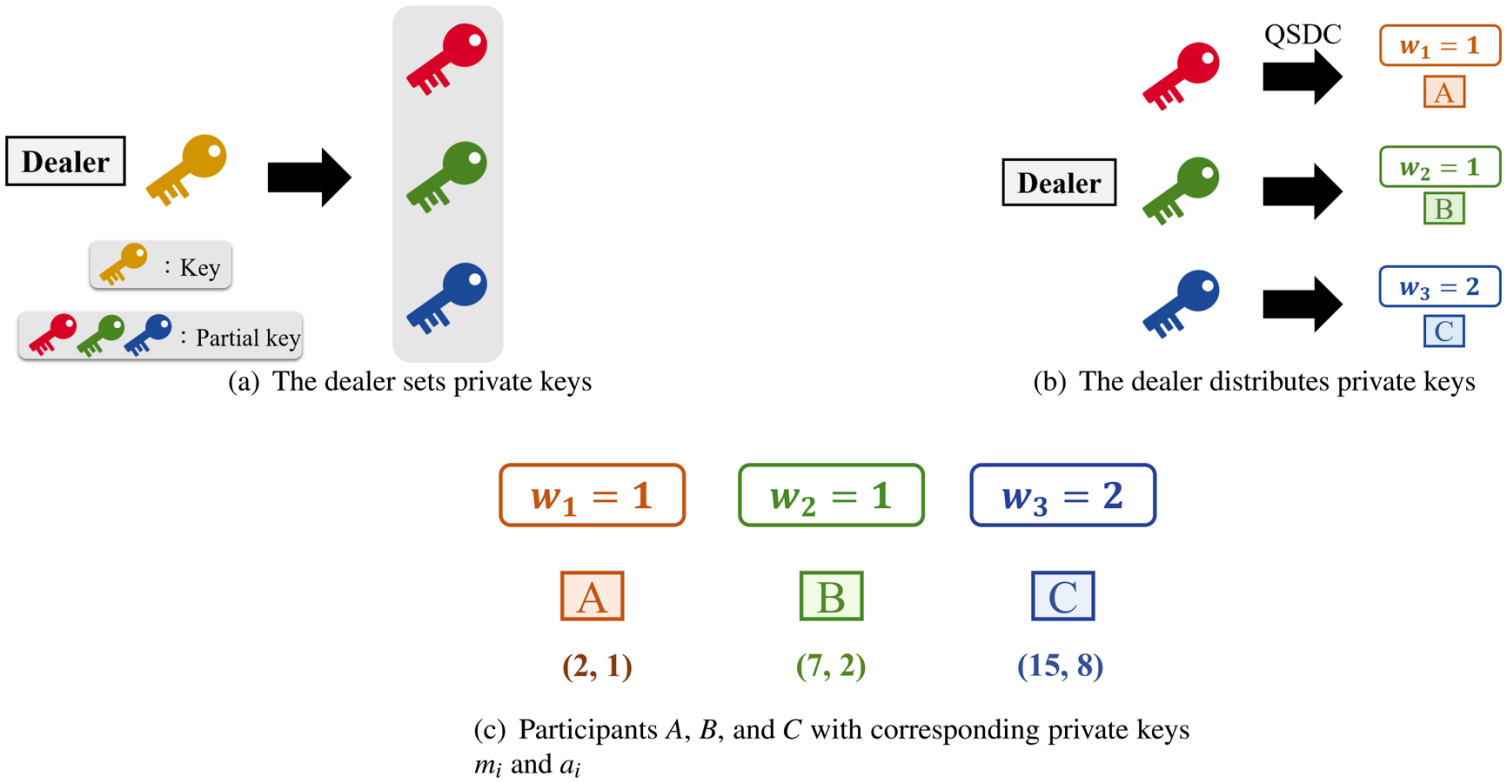
The dealer distributes private keys.

**Step 3. The dealer distributes private keys:** Depending on the corresponding positive integer $m_{i}$ the dealer prepares key *S*. If $m_{i}$ is taken as the divisor, the dealer will perform the formula to obtain remainder $a_{i}$ for all $1 \le i \le n$. Then, according to the weight $w _{i}$ of each participant $p_{i}$, the dealer will use QSDC^46^ to transfer the private partial keys $m_{i}$ and $a_{i}$ to corresponding participant $p_{i}$. For example, the dealer divides $S=23$ by $m_{1}=2$, to get $a_{1}=1$ and sends it to $p_{1}$, divides $S=23$ by $m_{2}=7$ to get $a_{2}=2$ and sends it to $p_{2}$, and divides $S=23$ by $m_{3}=15$ to get $a_{3}=8$ and sends it to $p_{3}$, as shown in Fig. 1.

**Figure 2:**
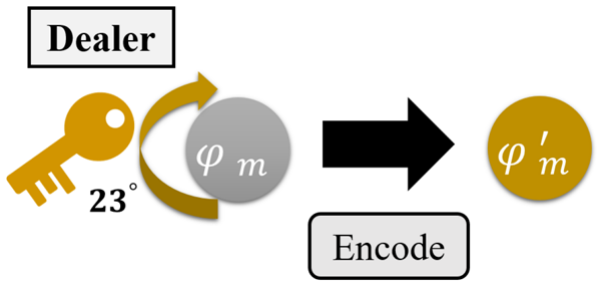
The dealer encrypts the quantum states.

**Step 4. The dealer uses the key to encrypt quantum particles: ** In this step, the dealer prepares *n* sequences $s_{1}$,$s_{2}$,$\ldots$,$s_{n}$ of unknown quantum states for the participants. The sequence is combined with $\{|\varphi _{1}\rangle =\alpha _{1} |0\rangle +\beta _{1} |1\rangle ,|\varphi _{2}\rangle =\alpha _{2} |0\rangle +\beta _{2} |1\rangle ,\ldots ,|\varphi _{m}\rangle = \alpha _{m} |0\rangle +\beta _{m} |1\rangle \}$. Then, the dealer rotates the quantum state which is performing the phase shift operation $U(\theta )$ in every quantum state to encrypt the quantum state. The example is shown in Fig. 2.

**Figure 3:**
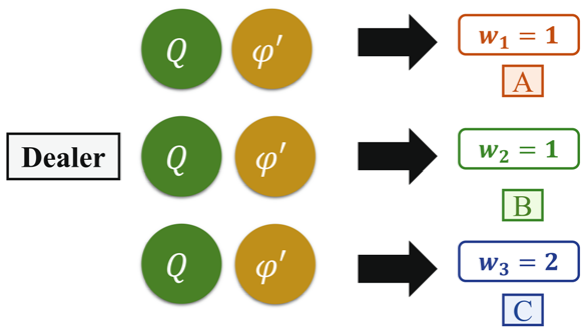
The dealer sends encrypted quantum particles and decoy particles.

**Figure 4:**
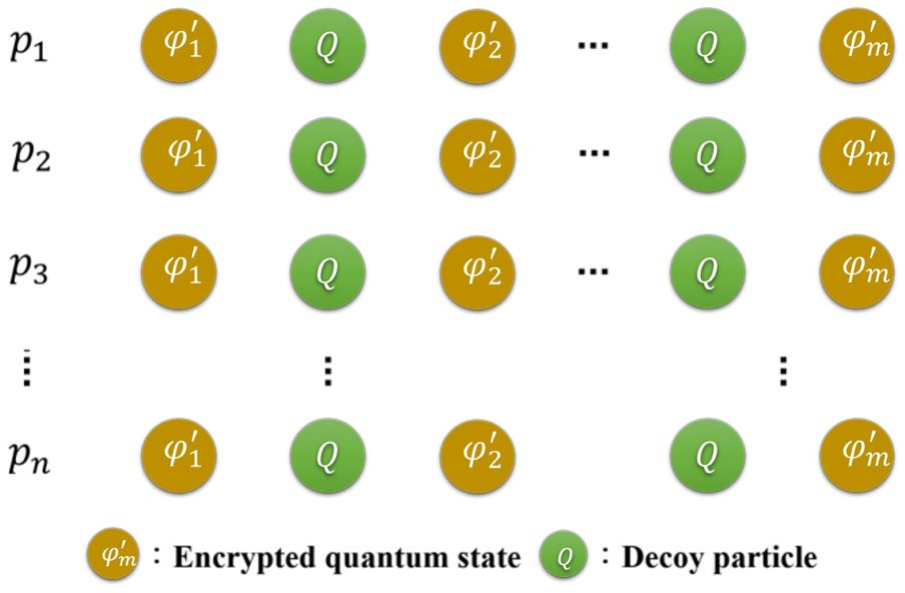
Schematic diagram of the encrypted quantum state and decoy particles.

**Step 5. Quantum channel:** The dealer randomly prepares a number of decoy particles in states $\{|0\rangle ,|1\rangle ,|+\rangle ,|-\rangle \}$, where $|+\rangle = \frac{1}{\sqrt{2}} (|0\rangle + |1\rangle )$ and $|-\rangle = \frac{1}{\sqrt{2}} (|0\rangle - |1\rangle )$), and then randomly inserts these decoy particles into *n* sequences, as shown in Fig. 4. The position and the initial state of each decoy particle is recorded, and the sequences $s_{1},s_{2},\ldots ,s_{n}$ are transferred to the corresponding participants $p_{1},p_{2},\ldots ,p_{n}$, as shown in Figs. 3 and 4. When all participants have received these sequences, the dealer will announce the position of the decoy particles publicly and ask the participants to measure these particles in the *Z*-basis or *X*-basis according to the basis that was sent. For example, when the dealer prepares the decoy particle $|0\rangle$ in the *Z*-basis to send to participants *A*, *B*, and *C*, the participants should measure the decoy particle to obtain the $|0\rangle$ with the *Z*-basis rather than $|1\rangle$. Similarly, when the dealer prepares the decoy particle $|+\rangle$ in the *X*-basis to send to the participants, the participant should measure the decoy particle to obtain the $|+\rangle$ with the *X*-basis rather than $|-\rangle$. Therefore, the dealer can calculate the error rate by comparing the measurement results to the initial states. If the error rate exceeds the threshold value, the dealer instructs the participants to abort the process and starts a new one from step 1. Otherwise, they continue to the next step.

**Figure 5:**
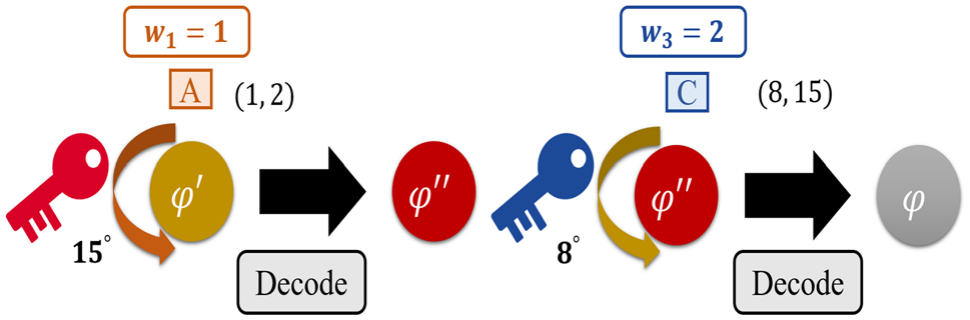
Simple diagram of *A* and *C* cooperating to reconstruct the secret.

**Step 6. The participants reconstruct the secret:** When the dealer finishes his or her job to securely send the quantum partial key, the dealer has completed the process of sharing the quantum particles. Then the participants will receive those quantum sequences. The criteria for reconstructing the secret is through the participant cooperation, that is when *t* of *n* participants decide to work together and their sum of weight should meet the fixed weighted threshold $\omega$. Assuming there are *t* participants $\{p_{1},p_{2},\ldots ,p_{t}\}$ who want to reconstruct the sequence. Every participant $p_{i}$ for all $1 \le i \le t$ have to use $m_{i}$ and $p_{i}$ which they have been sent during step 3 to calculate their own private partial key $S_{i}$ using CRT formula. Then, every participant $p_{i}$ should convert their own private partial key $S_{i}$ into radian $-\theta _{i}$ and performs the inverse phase shift operation $U(-\theta _{i})$ which is rotating the quantum state one by one on every quantum state in the sequence. Also, when participant $p_{i}$ delivers the quantum state in the sequence for next the participant $p_{i+1}$ they must similarly prepare a number of decoy particles and inserted them at random in states $\{|0\rangle ,|1\rangle ,|+\rangle ,|-\rangle \}$ in accordance with Step 5 for eavesdropping detection. After that, they can cooperate to decrypt the sequence which encrypted by key *S*. For example, participant *A* and participant *C* can cooperate to reconstruct the sequence encrypted by key *S*, which is equal to 23, because the sum of $w_{1}$, which is equal to 1, and $w_{3}$, which is equal to 2, is equal to a fixed weighted threshold $\omega$ equal to 3. Participant $p_{1}$ uses $m_{1}$ and $a_{1}$ to calculate their own private partial key $S_{1}$ as 15, and converts $S_{1}$ into radian $-\theta _{1}$. Participant $p_{3}$ uses $m_{3}$ and $a_{3}$ to calculate their own private partial key $S_{3}$ as 8, and converts $S_{3}$ into radian $-\theta _{3}$. They then perform the inverse phase shift operation $U(-\theta _{1})$ and $U(-\theta _{3})$ on every quantum state in the sequence, respectively. Finally, they can cooperate to reconstruct the sequence encrypted by key *S*, which is 23, to obtain the quantum information. A simple diagram is shown in Fig. 5.

### Security analysis

This section presents an analysis of the security of the proposed method. According to the way a key or message is intercepted, attacks are classified as either external attacks or internal attacks. In terms of external attacks, this study discusses whether an eavesdropper can steal the secret, or a lot of information without being detected. In terms of internal attacks, this study discusses whether a participant can reconstruct the secret alone, or participants can do when the weighted threshold requirement is not satisfied. Therefore, we will discuss some common types of attack as follows.

#### External attack

There are two common attacks: intercept-and-resend attacks and entangle-and-measure attacks. One discusses whether an eavesdropper can intercept the quantum state from the dealer and resend the new quantum state without detection. Another discusses whether an eavesdropper can use unitary operation $U_{e}$ to entangle a random particle on the decoy particles to steal information. These two common attacks can be defenced by decoy qubits^38^.

#### Intercept-and-resend attack

In step 5, before the dealer sends the quantum state sequences to the participants, they must randomly insert decoy particles in states $\{|0\rangle ,|1\rangle ,|+\rangle ,|-\rangle \}$ into the quantum state sequences. The dealer keeps a record of their positions and sends the sequences to the participants and asks them to measure these particles in the *Z*-basis or *X*-basis according to the basis that was sent and checks the measurement results with the participants. Since an eavesdropper will not know the position and state of the decoy particles, they will possibly measure them with the incorrect basis. Eavesdroppers will thus be detected with a probability of $1-(\frac{3}{4})^d$ for every decoy particle, where *d* is the number of decoy particles. When *d* is sufficiently large, the probability of detecting eavesdroppers will converge to 100%, thus ensuring absolute eavesdropper detection just like the detection rate in a quantum key distribution (BB84).

#### Entangle-and-measure attack

Although eavesdroppers can be detected in intercept-and-resend attacks, there is a possibility that they will use unitary operation $U_{e}$ to entangle a random particle on the decoy particles and measure the random particle in the *Z*-basis or the *X*-basis to steal the secret^39,40^. In the following, an eavesdropper performs unitary operation $U_{e}$ to entangle a particle $|E\rangle$ on the decoy particles in states $\{|0\rangle ,|1\rangle ,|+\rangle ,|-\rangle \}$.

2 $$\begin{aligned} \begin{aligned} U_e(|0\rangle |E\rangle )&=a|0\rangle |e_{00}\rangle +b|1\rangle |e_{01}\rangle \\ U_e(|1\rangle |E\rangle )&=c|0\rangle |e_{10}\rangle +d|1\rangle |e_{11}\rangle \\ U_e(|+\rangle |E\rangle )&=\frac{1}{\sqrt{2}}(a|0\rangle |e_{00}\rangle +b|1\rangle |e_{01}\rangle +c|0\rangle |e_{10}\rangle +d|1\rangle |e_{11}\rangle )\\&=\frac{1}{2}(|+\rangle (a|e_{00}\rangle +b|e_{01}\rangle +c|e_{10}\rangle +d|e_{11}\rangle )) +\frac{1}{2}(|-\rangle (a|e_{00}\rangle -b|e_{01}\rangle +c|e_{10}\rangle -d|e_{11}\rangle ))\\ U_e(|-\rangle |E\rangle )&=\frac{1}{2}(|+\rangle (a|e_{00}\rangle +b|e_{01}\rangle -c|e_{10}\rangle -d|e_{11}\rangle )) +\frac{1}{2}(|-\rangle (a|e_{00}\rangle -b|e_{01}\rangle -c|e_{10}\rangle +d|e_{11}\rangle )) \end{aligned} \end{aligned}$$

After the eavesdropper entangles $U_{e}$ to a particle $|E\rangle$ on the decoy particles, and obtains states $|e_{00}\rangle , |e_{01}\rangle , |e_{10}\rangle , |e_{11}\rangle$, $\left| a\right| ^2+\left| b\right| ^2+\left| c\right| ^2+\left| d\right| ^2 = 1$. In order to distinguish states $\{|0\rangle ,|1\rangle ,|+\rangle ,|-\rangle \}$ and avoid detection, the eavesdropper must set $b = 0$ and $c = 0$ to distinguish $|0\rangle$ or $|1\rangle$. This means that the eavesdropper can measure the state to obtain $|e_{00}\rangle$, deduce that its state is $|0\rangle$, measure the state to obtain $|e_{11}\rangle$, and then deduce that its state is $|1\rangle$. Then, they set $a-b+c-d=0$ and $a+b-c-d=0$ to distinguish $|+\rangle$ or $|-\rangle$. In order to satisfy both conditions, the result becomes $a-d = 0$. However, that result in a $a|e_{00}\rangle +b|e_{01}\rangle +c|e_{10}\rangle +d|e_{11}\rangle$ and $a|e_{00}\rangle -b|e_{01}\rangle -c|e_{10}\rangle +d|e_{11}\rangle$ becomes $a|e_{00}\rangle + d|e_{11}\rangle$. Therefore, eavesdropper will be unable to effectively distinguish $|e_{00}\rangle$ or $|e_{11}\rangle$ and will not get any useful information.

#### Internal attack

The condition to reconstruct the secret of $(w , \omega , n)$-weighted threshold quantum secret sharing schemes is that the total sum of the weights of whom the participants who want to cooperate, have to exceed a fixed weighted threshold $\omega$, and then they can recover the secret. However, if this requirement is not met, the secret cannot be reconstructed. That is because the maximum range of the key is decided by the minimum value from a set that achieves the weighted threshold, and the minimum range of the key is decided by the maximum value from a set that cannot achieve the weighted threshold. Therefore, the closer the sum of the weights is to the weighted threshold, the greater the possibility of the key being reconstructed. For example, in a ((1, 1, 2), 3, 3)-weighted threshold quantum secret sharing scheme, the respective weights of participants *A*, *B*, and *C* are $w_{1}=1$, $w_2={1}$, and $w_{3}=2$, and shares $m_{1}=2$, $m_{2}=7$, and $m_{3}=15$. If participants *A* and *B* want to cooperate to reconstruct the secret, and they can get a minimum positive integer of 9 by CRT, they can use the products of $m_{1}=2$ and $m_{2}=7$ to get the information ${23, 37, 51, \ldots , 9 + 14k}$ to perform the inverse phase shift operation, where $k \in {\mathbb {Z}}$. However, they will not know the range of the key, so they will not know how many products to use to perform the inverse phase shift operation to get key 23 and the quantum information.

Several excellent researchers recently propose studies^41–44^ about an internal attack on a multi-party quantum secret sharing protocol and their strategy to protect against it. The scenario of this kind of internal attack, as discussed in studies^41–44^, does not happen in our protocol, because the dealer distributes the parts of secrets to each participant individually by a secure quantum channel, such as QKD, and the participants do not need to distribute or forward information with each other. Once the distribution is finished, the dealer has no more information (nothing is left for stealing) and also does not need to anticipate the reconstruction process of the secret. Only the other participants have to cooperate with each other to reconstruct the secret. Only if the weights of the participants meet the threshold can the secret be reconstructed, and this is the basic operation principle in weighted threshold QSS. On the contrary, if there are not enough participants to cooperate, then they cannot rebuild the secret.

### Efficacy analysis

For quantum secret sharing protocols, a lower consumption of qubits is important to keep the cost is relatively low. Similarly, lower private key transmissions are significant, as this shows that the transmission effectiveness is better than others. Therefore, we will compare the proposed protocol with seven current protocols, namely, Cleve^13^, Tokunaga^14^, Qin^16^, Yang 1^45^, Yang 2^46^, Dehkordi^47^, and Li^17^.

There are two types of comparisons, according to different characteristics of the scenes. In order to test the efficiency of sharing information, we will analyze how many qubits each method costs. Therefore, in the same (*t*, *n*)-threshold scheme, we will compare the consumption of qubits for the same number of sharing bits. In order to test the efficiency of assigning the weight to the participants, we analyze how many private keys are transmitted for each method based on a single dimensional quantum state and how many qubits each method costs based on the multi-dimensional quantum state cost. We expand the threshold scheme to the weighted threshold scheme. In a $(w , \omega , {n})$-weighted threshold scheme, we compare the number of private key transmissions and the consumption of qubits for the same weight of a participant.

#### Consumption of qubits for same number of sharing bits

In the same threshold scheme, in order to compare the consumption of qubits with other protocols fairly in the same number of sharing bits, we will calculate how many qubits are spent in sharing *N* bits for each protocol. The following is an analysis for Tokunaga^14^, Qin^16^, Yang_1^45^, Yang_2^46^, and Dehkordi^47^.

**Tokunaga**^14^ **and Qin**^16^: In the threshold scheme, in order to share *N* bits, the dealer prepares the *N* qubits for each participant.

**Yang1**^45^: In order to share *N* bits, the dealer prepares the *N* qubits and 2*N* bell state for each participant.

**Yang2**^46^: In order to share *N* bits, the dealer prepares the 2*N* bell state for each participant.

**Dehkordi**^47^: In order to share *N* bits, the dealer prepares the 3*N* GHZ state for each participant.

**Figure 6:**
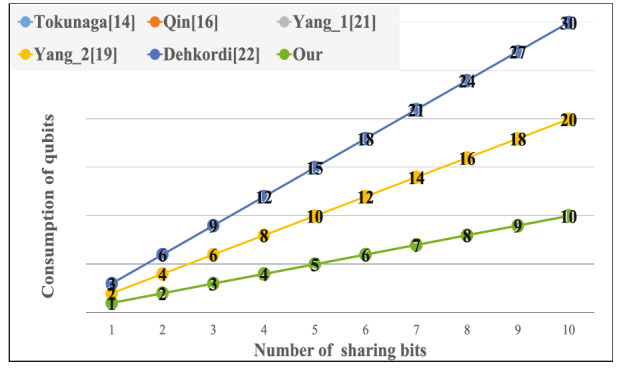
Comparison of the consumption of qubits for the same number of sharing bits.

First, we compare the consumption of qubits for the same number of sharing bits in the same (*t*, *n*)-threshold secret sharing scheme and test whether our method is better than the other protocols based on a single dimensional quantum state as shown in Fig. 6 and Table 1. When the number of sharing bits increases, the consumption of quantum resources is several times the sharing bits for the other protocols, but the proposed method is the same as the number of shared bits.

**Table 1:** The cost of single dimensional quantum state.

	Consumption of qubit/sharing bit	Private key transmission/weight
Tokunaga^14^	N	M
Qin^16^	N	M
Yang_1^45^	3N	M
Yang_2^46^	2N	M
Dehkordi^47^	3N	M
Our	N	1

**Table 2:** The cost of multi-dimensional quantum state.

	Consumption of qubit/weight
Cleve^13^	2K
Li^17^	K
Our	1

#### Private key transmission and consumption of qubit for same weight

In order to compare the number of private key transmissions in a single dimensional quantum and the consumption of qubits in a multi-dimensional quantum state for the same weight of a participant, we expand the threshold scheme to the weighted threshold scheme. Then, for each protocol, we calculate how many private key transmissions they transfer for *M* weights of the participants, and how many qubits are consumed for *K* weight of participants. The following is an analysis for Cleve^13^, Tokunaga^14^, Qin^16^, Yang_1^45^, Yang_2^46^, and Dehkordi^47^, Li^17^.

**Tokunaga**^14^, **Qin**^16^, **Yang**_**1**^45^, **Yang**_**2**^46^ **and Dehkordi**^47^: In the weighted threshold scheme, the dealer uses Lagrange Interpolation to transmit *M* private keys for each participant according to the weight of the participant.

**Cleve**^13^: The dealer utilizes the 2*K* multi-dimensional quantum state to express Lagrange Interpolation for each participant according to the weight of the participant.

**Li**^17^: The dealer utilizes the *K* multi-dimensional quantum state to express Lagrange Interpolation for each participant according to the weight of the participant.

**Figure 7:**
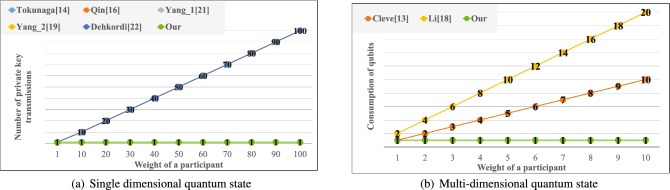
Comparison of the number of private key transmissions and the consumption of qubits for the same weight of a participant.

Next, because the proposed method is based on the CRT and phase shift operation, we can build not only a (*t*, *n*)-threshold secret sharing scheme but also a $(w , \omega , n)$-weighted threshold scheme. In order to fairly test, we will extend the other protocols based on a single dimensional or multi-dimensional quantum state to a $(w , \omega , n)$-weighted threshold scheme to compare the number of private key transmissions and the consumption of qubits for same weight of a participant, as shown in Fig. 7, Tables 1 and 2.

According to result of Fig. 7a, when the weight of a participant increases, the demand for the private key increases for the other protocols based on a single dimension, and the proposed method only needs one. According to result of Fig. 7b, when the weight of a participant increases, the consumption of quantum resources has increases drastically for the other protocols based on multiple-dimension, and the consumption of quantum resources of the proposed method still remain one.

## Discussion

Most proposed quantum threshold schemes are based on a multi-dimensional quantum state and Lagrange Interpolation which are too complicated to implement practically. The proposed method is different from traditional method and it based on the CRT and phase shift operation. The reason we use CRT is that the characteristic of CRT dividing and recovery offers a simple and efficient way to make partial keys/ shares. The reversibility of phase shift operation can encode and decode a secret on quantum bits to share quantum information. In the proposed weighted threshold QSS method, the dealer is able to decide the key and encode the key in a quantum bit using the phase shift operation, divide the key into a partial key to be shared using CRT, and then using QSDC to send these partial keys to all participants as their private keys. To reconstruct the secret does not necessary to have all participants. When some participants want to cooperate and reconstruct the secret and the criteria is that their sum of the weights have to exceed a fixed weighted threshold $\omega$. Then, participants can use their own private key to perform inverse phase shift operations on the quantum states one by one to decode the quantum states. After that, participants can obtain the original secret which is the quantum information back. This study has three major contributions. First, the proposed weighted threshold QSS method is flexible, unconditionally secure cryptosystem, and easy to implement. Second, most traditional QSS schemes share classical information, while the proposed method is the first attempt to share quantum information. Third, the proposed scheme requires lower resources than other protocols, as we using single quantum particles rather than using multi-dimension quantum states which the previous methods do, making our method more efficient.

## Data Availability

No datasets were generated or analysed during the current study.
